# The FAM86 domain of FAM86A confers substrate specificity to promote EEF2-Lys525 methylation

**DOI:** 10.1016/j.jbc.2023.104842

**Published:** 2023-05-18

**Authors:** Joel William Francis, Zengyu Shao, Pradnya Narkhede, Annie Truc Trinh, Jiuwei Lu, Jikui Song, Or Gozani

**Affiliations:** 1 Department of Biology, Stanford University, Stanford, California, USA; 2 Department of Biochemistry, University of California, Riverside, California, USA

**Keywords:** alphafold, EEF2, EEF2KMT, FAM86A, lysine methylation, methyltransferase, mRNA translation, protein synthesis, ribosome, translation elongation

## Abstract

FAM86A is a class I lysine methyltransferase (KMT) that generates trimethylation on the eukaryotic translation elongation factor 2 (EEF2) at Lys525. Publicly available data from The Cancer Dependency Map project indicate high dependence of hundreds of human cancer cell lines on FAM86A expression. This classifies FAM86A among numerous other KMTs as potential targets for future anticancer therapies. However, selective inhibition of KMTs by small molecules can be challenging due to high conservation within the *S-*adenosyl methionine (SAM) cofactor binding domain among KMT subfamilies. Therefore, understanding the unique interactions within each KMT–substrate pair can facilitate developing highly specific inhibitors. The FAM86A gene encodes an N-terminal FAM86 domain of unknown function in addition to its C-terminal methyltransferase domain. Here, we used a combination of X-ray crystallography, the AlphaFold algorithms, and experimental biochemistry to identify an essential role of the FAM86 domain in mediating EEF2 methylation by FAM86A. To facilitate our studies, we also generated a selective EEF2K525 methyl antibody. Overall, this is the first report of a biological function for the FAM86 structural domain in any species and an example of a noncatalytic domain participating in protein lysine methylation. The interaction between the FAM86 domain and EEF2 provides a new strategy for developing a specific FAM86A small molecule inhibitor, and our results provide an example in which modeling a protein–protein interaction with AlphaFold expedites experimental biology.

Protein lysine methylation is the addition of one, two, or three methyl moieties to the ε-nitrogen of a lysine side chain, forming mono-, di-, and trimethylated derivatives (1). Lysine methylation is an abundant posttranslational modification in humans, carried out by dozens of lysine methyltransferases (KMTs) encoded in the human genome (1, 2, 3). Human KMTs belong to one of two large families: SET (Su(var)3–9, Enhancer-of-zeste, Trithorax) domain enzymes and 7βS (seven-β-strand) domain enzymes (4). Members of the SET domain family are known to generate lysine methylation on histones as well as nonhistone proteins and serve both nuclear and cytoplasmic functions (5). Members of the 7βS family, with few exceptions (6, 7), generally methylate nonhistone proteins and serve cytoplasmic activities (3). Human KMTs regulate diverse biological processes via their methylation activities, including the fundamental processes of transcription and translation (1, 4, 5). Accordingly, dysregulation of many KMTs, for example by aberrant expression or mutation, has been linked to diverse human diseases including cancer (1, 2, 5). FAM86A is a member of the 7βS family known to generate trimethylation on lysine 525 (K525me3) on the eukaryotic translation elongation factor 2 (EEF2) (8, 9).

EEF2 performs the essential function of facilitating ribosomal translocation during mRNA translation (10, 11, 12, 13). It has been reported that translational control of the proteome, including EEF2 upregulation (14, 15), is common in human cancers (16, 17). Notably, publicly available data from The Cancer Dependency Map project (DepMap) indicate high dependence of hundreds of human cancer cell lines on expression of the EEF2 methyltransferase FAM86A (Fig. S1) (18, 19). This observation suggests there may be value in targeting FAM86A for oncological indications. While there has been success in the last decade in developing selective small molecule KMT inhibitors, advancing inhibitors for many KMTs into successful clinical trials is an ongoing challenge (1, 20, 21). Many small molecule inhibitors under investigation for clinical use are competitors of *S*-adenosylmethionine (SAM), the methyl donor in lysine methylation catalysis (1, 20). While inhibiting KMTs with SAM-competitive molecules can be effective, these molecules carry the challenge of potential poor selectivity since all KMTs utilize SAM for catalysis. Thus, identifying unique characteristics within KMT–substrate pairs can aid in the development of specific KMT inhibitors.

It is common for KMTs to encode structural domains in addition to catalytic domains (*e.g.*, (22)). In both *Saccharomyces cerevisiae* and humans, FAM86A encodes an uncharacterized FAM86 domain as well as its catalytic methyltransferase (MTase) domain (Fig. 1*A*). Primary amino acid sequence alignment shows the FAM86 and MTase domains of FAM86A are conserved from yeast with 20% and 28% conserved identity, respectively (Fig. 1*A*). Although the FAM86 domain evolved alongside the MTase domain through the evolutionary tree, there remains no known biological function for the FAM86 domain in any species.

**Figure 1:**
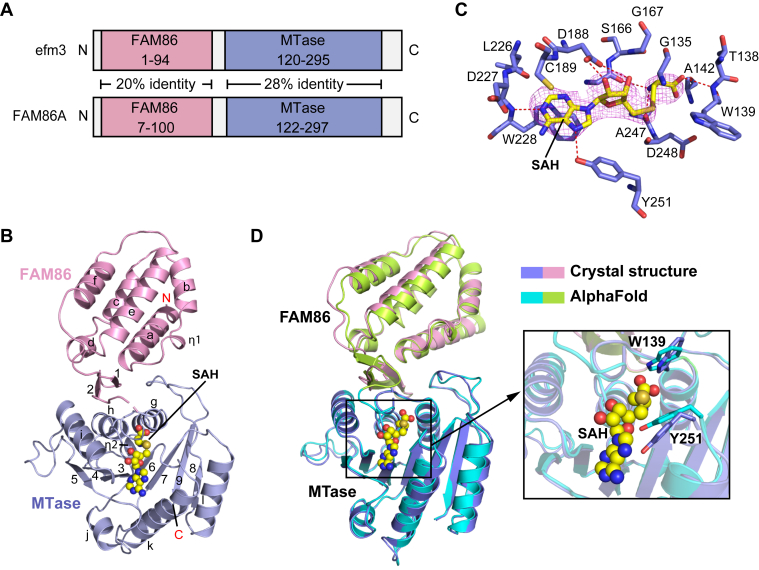
**FAM86A structural models determined by X-ray crystallography and AlphaFold.***A*, schematic of yeast efm3 and human FAM86A proteins domain structures. *B*, ribbon representation of human FAM86A bound to *S*-adenosyl homocysteine (SAH) as determined by X-ray crystallography. *C*, close-up view of the interaction between human FAM86A and SAH, with hydrogen-bonding interactions depicted as dashed lines. The Fo–Fc omit map (*magenta*) of SAH is contoured at 2.5σ level. *D*, superimposition of FAM86A X-ray crystallography and AlphaFold predicted structures.

AlphaFold is the machine learning algorithm developed by DeepMind to predict protein structures based on primary amino acid sequences (23). In the last couple of years, hundreds of thousands of protein models have been predicted by AlphaFold and made available to the public (24). These models are often highly accurate based on comparisons with experimentally determined structures (23, 25). AlphaFold-Multimer is a new iteration of AlphaFold designed to predict possible conformations of multichain protein complexes (26). Therefore, the simulation of protein structures and protein–protein interactions *in silico* has the potential to facilitate the process of experimental biology by enabling molecular insights on an accelerated timescale. In this work, we used a combination of X-ray crystallography, the newest AlphaFold algorithms, and experimental biochemistry to characterize the role of the FAM86 domain in EEF2 methylation by FAM86A.

Using the structural data provided by X-ray crystallography and AlphaFold, we determined the FAM86 domain of FAM86A forms a five-helix bundle distinct from the 7βS domain. By testing the catalytic activity of a truncated FAM86A construct lacking the FAM86 domain, we found the FAM86 domain is required for EEF2 methylation *in vitro* and in human cells. Simulated models of the FAM86A–EEF2 interaction generated by AlphaFold-Multimer suggested the FAM86 domain of FAM86A forms an extensive intermolecular interaction with EEF2. Point mutations within this predicted interface inhibited EEF2 methylation in human cells. Based on these data, we propose that the FAM86 domain of FAM86A confers substrate specificity by orienting EEF2-Lys525 toward the FAM86A active site. Future work may leverage these insights or take similar approaches to develop selective inhibitors of FAM86A and other KMTs.

## Results

### Characterization of FAM86A crystal structure and AlphaFold model

Our initial effort in crystallizing wildtype FAM86A failed to generate diffractable crystals. To overcome this challenge, we designed a FAM86A construct harboring a triple mutation for reduction of surface entropy (see Experimental procedures), which permitted structure determination of full-length FAM86A in complex with cofactor–by-product *S*-adenosyl homocysteine (SAH) at 3.3-Å resolution (Figs. 1, *B* and *C*, S2 and Table 1). The crystal structure of the FAM86A–SAH complex belongs to the I4_22_ space group, containing three complexes in one asymmetric unit (Fig. S2*A*). We were able to trace the entire FAM86A protein, except for the very N terminus (residues 1–5) and a portion of the domain linker between the FAM86 and MTase domains (residues 127–133).

**Table 1:** Crystallographic data collection and refinement statistics

	FAM86–SAH (PDB 8FZB)
Data collection	
Space group	*I 4 2 2*
Cell dimensions	
*a*, *b*, *c* (Å)	160.7, 160.7, 351.8
α, β, γ (°)	90, 90, 90
Wavelength	0.9792
Resolution (Å)	48.72–3.33 (3.45–3.33)^a^
*R*_merge_	0.149 (1.64)
*I*/σ*I*	19.8 (1.8)
CC_1/2_	0.999 (0.359)
Completeness (%)	98.6 (96.6)
Redundancy	39 (34)
Total reflections	1,324,854 (112,423)
Unique reflections	33,996 (3229)
Refinement	
No. of reflections	33,573
*R*_work_/*R*_free_ (%)	23.0/25.3
No. of atoms	
Protein	7300
Ligands	78
*B* factors (Å^2^)	
Protein	139.8
Ligands	104.5
RMS deviations	
Bond lengths (Å)	0.003
Bond angles (°)	0.67
Ramachandran	
Favored (%)	96.85
Allowed (%)	3.15
Outliers (%)	0

a Values in parentheses are for the highest-resolution shell. The dataset was collected from a single crystal.

The structure of FAM86A reveals a two-lobe architecture, in which the N-terminal FAM86 domain stacks right on top of the C-terminal methyltransferase domain, creating a potential catalytic cleft commonly observed for DNA methyltransferases (Fig. 1, *B* and *C*) (27, 28). The FAM86 domain is dominated by a five-helix bundle (αa, αb, αc, αe, and αf), which packs against a following two-stranded (β1 and β2) β-sheet via αa- and intervening αd-helix. The methyltransferase domain assumes a Rossmann fold, in which a seven-stranded central β-sheet is flanked by three α-helices on both sides.

We next examined the predicted model of FAM86A’s 3D structure available through the AlphaFold database with ligand transposition provided by AlphaFill (29). The predicted model bears striking resemblance to the experimental crystal structure, with root-mean-square deviation (RMSD) of 1.2 Å over 319 aligned Cα atoms (Fig. 1*D*). Of note, the experimental and predicted models reveal the nearly identical SAM-binding pocket with approximately 14 residues participating in the coordination of the ligand SAH (Fig. 1*D*) Nevertheless, the experimental and predicted models show a slight difference in the helical orientations (αe and αf) in the FAM86 domain, as well as the positionings of the SAH-engaging residues W139 and Y251 (Fig. 1*D*). AlphaFill did not predict any ligands to interact with the FAM86 domain.

### The FAM86 domain of FAM86A is required for EEF2-Lys525 methylation

**Figure 2:**
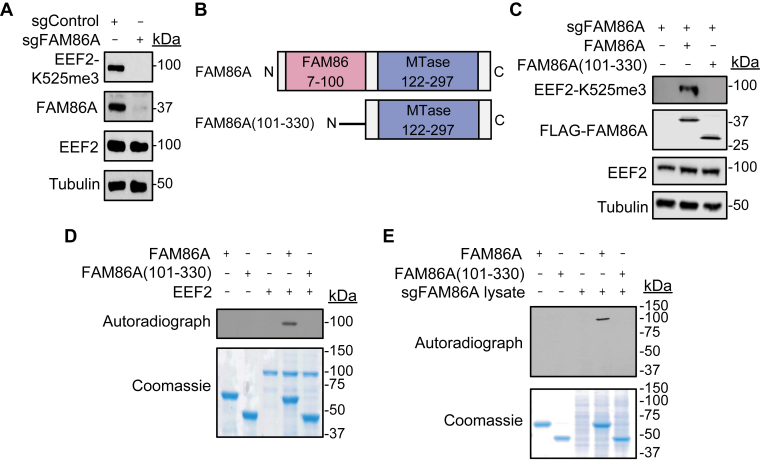
**The FAM86 domain is required for EEF2 methylation by FAM86A.***A*, Western blot analysis of whole cell extracts from control or FAM86A-depleted HEK293T cells. *B*, schematic of wildtype FAM86A and FAM86A(101–330) lacking the FAM86 domain. *C*, Western blot analysis of whole cell extracts from FAM86A-depleted HEK293T cells complemented with the indicated constructs described in (*B*). *D*, *in vitro* methylation assay with recombinant wildtype or truncated FAM86A as indicated with recombinant EEF2 as substrate. Top panel, ^3^H-SAM is the methyl donor and methylation visualized by autoradiography. Bottom panel, Coomassie blue stain of the proteins in the reaction. *E*, *in vitro* methylation assay as in (*D*) using whole cell extracts from FAM86A-depleted HEK293T cells shown in (*A*) as the substrate.

We began our investigation into FAM86A methylation by raising an antibody that is highly selective and specific for EEF2-K525 trimethylation (Fig. S3). First, we generated FAM86A knockout (KO) HEK293T cells and found endogenous EEF2-K525me3 levels were completely depleted in the KO cells (Fig. 2*A*). We used the custom antibody to ask whether the FAM86 domain of FAM86A is required for EEF2 methylation. To test this, we cloned a truncated construct of FAM86A (FAM86A(101–330)) lacking the FAM86 domain (Fig. 2*B*) and tested its activity on EEF2 by adding back wildtype or truncated FAM86A to KO cells by transfection. While wildtype FAM86A generated EEF2-K525me3, the truncated mutant, which expressed at the same level as wildtype protein, did not (Fig. 2*C*). Next, we asked a similar question *in vitro* by incubating recombinant, purified FAM86A with recombinant, purified EEF2 in the presence of radiolabeled SAM (^3^H-SAM). Consistent with the result in cells, we found the FAM86 domain is required for EEF2 methylation *in vitro* (Fig. 2*D*). When recombinant, purified FAM86A(101–330) was incubated with whole cell extracts from the FAM86A KO HEK293T cells *in vitro*, we did not observe activity on any protein, whereas full-length protein methylated a protein the size of EEF2 (Fig. 2*E*). From these data we conclude the FAM86 domain is required for EEF2-Lys525 methylation by FAM86A and that EEF2 is the likely main physiologic target of FAM86A.

### Simulation of the FAM86A–EEF2 interface by AlphaFold-Multimer

**Figure 3:**
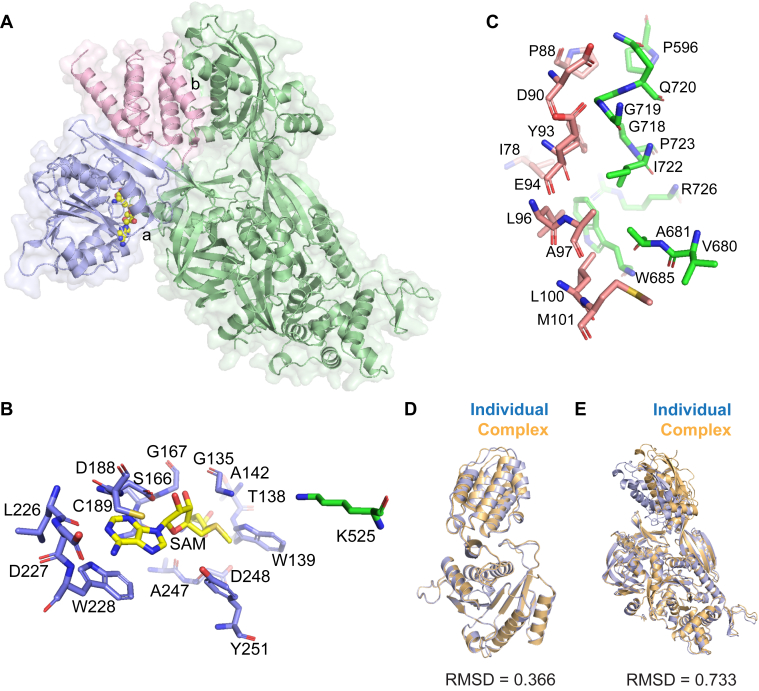
**Simulation of the FAM86A–EEF2 interface by AlphaFold-Multimer.***A*, ribbon representation of the overall complex predicted by AlphaFold-Multimer. EEF2 is colored in *green*. FAM86A is colored in *blue* for the MTase domain and *pink* for the FAM86 domain. *S-*adenosyl methionine (SAM) is shown as spheres. Spaces containing intermolecular interaction within 3 Å are designated as (a) near the FAM86A active site and (b) for the interaction involving the FAM86 domain of FAM86A and domain IV of EEF2. *B*, close-up view of (a) showing the proximity of EEF2-Lys525 to the SAM-binding pocket of FAM86A. *C*, close-up view of (b) showing the intermolecular interaction between the FAM86 domain of FAM86A and domain IV of EEF2. *D* and *E*, overlays of FAM86A (*D*) and EEF2 (*E*) individual AlphaFold models superimposed onto the FAM86A–EEF2 complex from AlphaFold-Multimer. Models of FAM86A and EEF2 determined individually are shown in *blue*, and models determined in complex with one another are shown in *orange*.

We next considered whether the FAM86 domain participates in the physical interaction between FAM86A and EEF2. To test this *in silico*, we queried AlphaFold-Multimer via ColabFold (30) to simulate a heterodimeric model containing the FAM86A and EEF2 proteins in a 1:1 stoichiometry. AlphaFold-Multimer outputs multiple models containing the possible conformations of the queried protein sequences in complex with each other, assigning a confidence ranking to each model. The models for our query were all nearly identical to one another, and the model designated with the highest confidence ranking by AlphaFold-Multimer is analyzed here. A physical interaction of less than 3 Å was predicted, involving both the FAM86 and MTase domains of FAM86A (Fig. 3*A*). Importantly, EEF2-Lys525 was predicted to occupy a position proximal to the FAM86A active site near the SAM-binding residues that were independently identified by X-ray crystallography and AlphaFill (Fig. 3*A*). When superimposed with the AlphaFill model, we can visualize the proximity of EEF2-Lys525 to the ligand SAM as if FAM86A, EEF2, and SAM were experimentally cocrystallized (Fig. 3*B*). In a second interface, an interaction was predicted involving the FAM86 domain and domain IV of EEF2 (Fig. 3*A*). A close-up view reveals an extensive network of interactions involving FAM86A residues Ile78, Pro88, Asp90, Try93, Glu94, Leu96, Ala97, Leu100, and Met101 as well as EEF2 residues Pro596, Val680, Ala681, Trp685, Gly718, Gly719, Gln720, Ile722, Pro723, and Arg726 (Fig. 3*C*). Since AlphaFold-Multimer folds proteins with a goal of simulating an interaction, we superimposed the models of FAM86A and EEF2 that were individually predicted by AlphaFold onto the FAM86A–EEF2 complex as a measure of quality control. We found the individual FAM86A model was nearly identical to its conformation in the FAM86A–EEF2 complex, with RMSD of only 0.366 Å over 252 aligned Cα atoms (Fig. 3*D*). In the case of EEF2, the RMSD was calculated to be 0.733 Å over 642 aligned Cα atoms (Fig. 3*E*). Most of the variation for EEF2 is caused by rotation of domain IV, a physiologic flexibility known to be crucial for GTP hydrolysis and ribosomal translocation (12, 31). Given the high degree of similarity with each protein’s individual model, we viewed the predicted FAM86A–EEF2 model as a reasonable basis for further experiments. Therefore, we hypothesized that the predicted FAM86A–EEF2 complex accurately portrays an interaction between the FAM86 domain of FAM86A and domain IV of EEF2 that orients EEF2-Lys525 toward the FAM86A active site.

### Allosteric inhibition of EEF2 methylation by point mutation of FAM86A and EEF2

**Figure 4:**
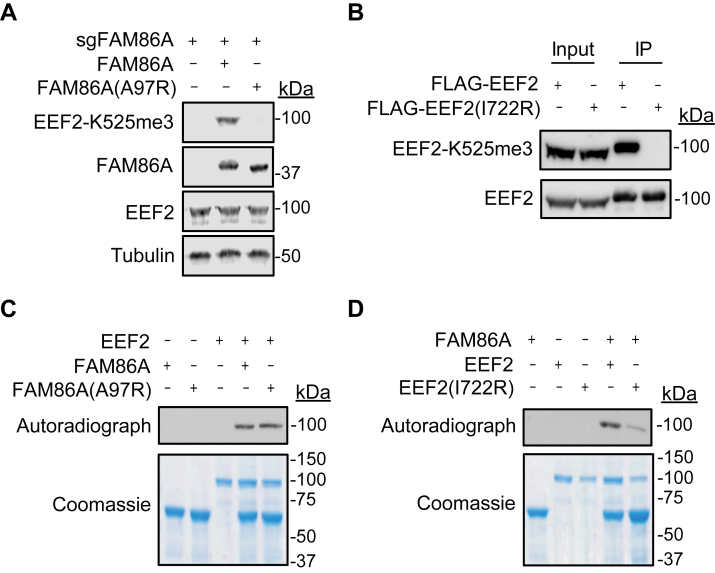
**Allosteric inhibition of EEF2 methylation by point mutation of FAM86A and EEF2.***A*, Western blot analysis of whole cell extracts from FAM86A-depleted HEK293T cells complement with either wildtype or A97R-substituted FAM86A. *B*, FLAG immunoprecipitation of either wildtype or I722R-substituted EEF2 from HEK293T cells. *C* and *D*, *in vitro* methylation assays as in Figure 2 with the indicated combinations.

To test the AlphaFold-Multimer predicted interaction, we asked if blocking the interaction between the FAM86 domain of FAM86A and domain IV of EEF2 inhibits FAM86A-mediated EEF2-Lys525 methylation. We generated model-guided FAM86A and EEF2 derivatives carrying point mutations in the FAM86 domain and domain IV, respectively. Specifically, FAM86A-Ala97 and EEF2-Ile722 were substituted to arginine to increase the likelihood of disrupting the binding interface while still maintaining the native individual structures of FAM86A and EEF2 (Fig. S4, *A*–*D*). As shown in Figure 4*A*, complementation of FAM86A (A97R) in FAM86A KO HEK293T cells failed to rescue EEF2-Lys525 methylation in cells, whereas wildtype FAM86A restored methylation (Fig. 4*A*). To test whether EEF2(I722R) is a viable substrate for FAM86A, we transfected and immunoprecipitated FLAG-EEF2(I722R) from HEK293T cells endogenously expressing FAM86A. While the input sample shows methylation of endogenous wildtype EEF2 in the I722R sample, the exogenous EEF2 carrying I722R substitution was not methylated (Fig. 4*B*). Importantly, both FAM86A (A97R) and EEF2(I722R) mutant constructs expressed equally well compared with wildtype in cells and are predicted by AlphaFold to not impact overall protein folding (Figs. 4, *A* and *B* and S4, *C* and *D*). These data suggest that substitutions of FAM86A (A97R) and EEF2(I722R) prevent EEF2-Lys525 methylation through an allosteric mechanism, likely due to inhibition of the interface predicted by AlphaFold-Multimer.

*In vitro*, the activity of purified FAM86A (A97R) on purified EEF2 was similar to that of wildtype FAM86A (Fig. 4*C*). In contrast, purified FAM86A showed reduced activity on purified EEF2(I722R) compared with wildtype EEF2 (Fig. 4*D*). We postulate that, in a physiological context, the transient nature of the FAM86A and EEF2 interaction becomes sensitive to the modest interference generated by the point mutations. Meanwhile, the simplicity and high stoichiometry of each protein in *in vitro* reaction mixtures amplify the possibility that any two proteins might interact with one another.

**Figure 5:**
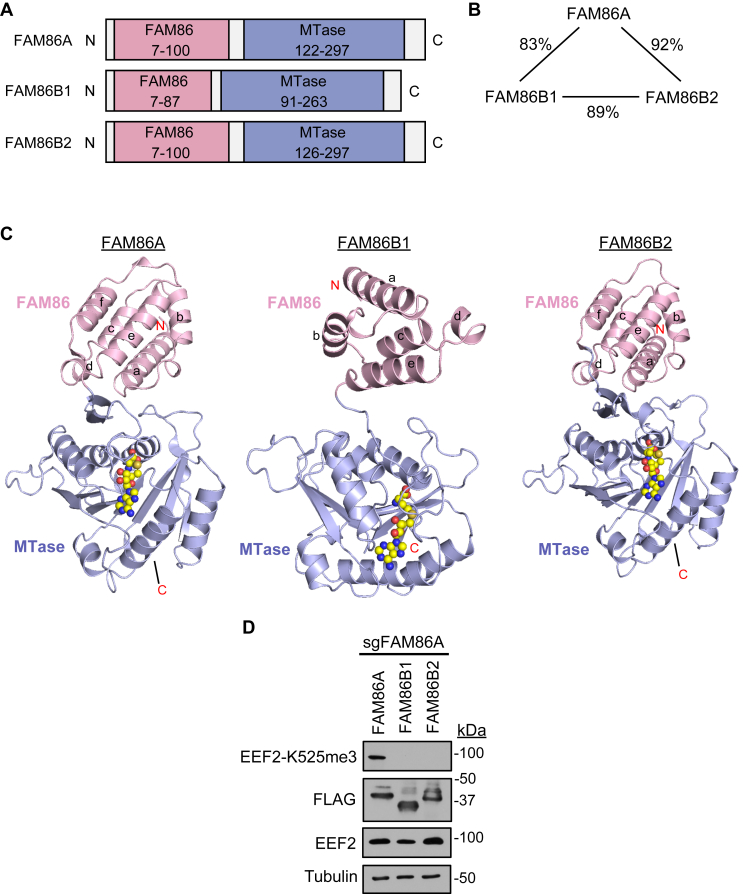
**Examination of other FAM86 domain–containing KMTs**. *A*, domain structures of FAM86A, FAM86B1, and FAM86B2. *B*, schematic representation of homology between FAM86A, FAM86B1, and FAM86B2. Percentages indicate identical amino acid residues conserved between proteins. *C*, ribbon representations of AlphaFold models for FAM86A (*left*), FAM86B1 (*middle*), and FAM86B2 (*right*) colored in *blue* for MTase domains and *pink* for FAM86 domains. *D*, Western blot analysis of whole cell extracts from FAM86A-depleted HEK293T cells transfected with FAM86A, FAM86B1, or FAM86B2.

The implication that the FAM86 domain of FAM86A confers substrate specificity for EEF2 is intriguing on multiple fronts. First, this is an instance in which a noncatalytic domain of a KMT is required for catalysis. Second, the FAM86 domain represents a novel and clinically actionable target for inhibition of FAM86A catalytic activity, which may be relevant given the possible role of the FAM86A–EEF2 methylation axis in cancer biology. Third, there are other FAM86 domain–containing human KMT genes that likely emerged from FAM86A as a common ancestor. While FAM86A exists in yeast, neither of the human genes FAM86B1 or FAM86B2 exists in many lower animals including mice (32). FAM86B1 and FAM86B2 are both uncharacterized members of the class I methyltransferase family with no known substrates. Alignment of FAM86A, FAM86B1, and FAM86B2 reveals nearly identical homology between the three genes, implying a close evolutionary relationship (Fig. 5, *A* and *B*). FAM86B2 is nearly identical to FAM86A, while FAM86B1 encodes a truncated FAM86 domain resulting from loss of a single exon compared with FAM86A and FAM86B2 (33). While the FAM86 domains of FAM86A and FAM86B2 form five-helix bundles, the FAM86 domain of FAM86B1 only forms a four-helix bundle (Fig. 5*C*). Notably, the exon lost in FAM86B1 encodes the homologous helix that forms the interface of FAM86A with EEF2. We tested whether FAM86B1 and FAM86B2 are redundant copies of FAM86A by adding back FAM86B1 and FAM86B2 to FAM86A KO HEK293T cells. However, neither FAM86B1 nor FAM86B2 generates EEF2-K525me3 (Fig. 5*D*), suggesting that they may have different substrates or not be active enzymes.

## Discussion

FAM86A was previously reported to methylate EEF2-Lys525 in yeast and humans. Here we provide the first report of a function for the FAM86 domain in EEF2 methylation or in any other biological process. Given the extremely high homology between FAM86A and FAM86B2, we suspect but cannot conclude that FAM86B2 is an inactive duplicate of FAM86A in humans. The DepMap gene effect data support this hypothesis by revealing no dependence of human cells on FAM86B2 expression, in contrast with high dependence on FAM86A (Figs. S1 and S5*A*). However, FAM86B1 is categorized as an essential gene by its DepMap gene effect data (Fig. S5*B*). This suggests that it could be an active enzyme in human cells and hints at an interesting evolutionary story: by deleting the residues of its FAM86 domain that promote EEF2-Lys525 methylation, FAM86B1 evolved specificity for a different substrate that, like the FAM86A–EEF2 methylation axis, promotes a critical function in human cells. A future iteration of AlphaFold (34) could serve as the tool by which to identify the substrate of FAM86B1.

This study describes the use of artificial intelligence to aid experimental biology. While direct truncation experiments could have been used to determine the FAM86 domain role in EEF2 methylation by FAM86A, the use of AlphaFold provided clear and testable molecular hypotheses. For example, it could have been laborious to identify the FAM86-binding residues of EEF2 without the information provided by structural modeling. In our case, it took less than 1 month to progress from hypothesis to simulated models to experimental results. This study supports the potential of AlphaFold algorithms to accelerate the process of experimental biology toward enhancing our understanding of biology and human disease.

## Experimental procedures

### Cell lines

HEK293T (female, embryonic kidney) cells were grown in Dulbecco’s modified Eagle’s medium supplemented with 10% fetal bovine serum and 100 U/ml penicillin/streptomycin. Cells were cultured at 37 °C in a humidified incubator with 5% CO2. Cell lines were authenticated by short tandem repeat profiling and tested negative for mycoplasma (DDC Medical).

### Transfection and viral transduction

Transient expression was performed using polyethylenimine (PEI) 3 μg per 1 μg of plasmid DNA. For CRISPR-Cas9 knockout of FAM86A, virus particles were produced by cotransfection of 293T cells with the lentiCRISPR v2/puro (Addgene) construct containing the sgRNA sequence AGCACGGCCATCATCTCCTA, pCMV-VSV-G (Addgene), and pCMV-dR8.2 dvpr (Addgene) in a ratio of 5:1:4 by mass. As a control, viruses were prepared in the same manner using the safe-targeting sgRNA sequence GGGCTACTAGGATTCAATCT (35). The medium was changed 24 h after transfection. After 48 h, target cells were transduced with 0.45 μm filtered viral supernatant and 4 μg/ml polybrene. The medium was changed 24 h after transduction. Cells were selected with 2 μg/ml puromycin beginning 48 h after transduction and continuing for 7 days.

### Plasmids

In addition to plasmids listed for virus production, the following plasmids were cloned and used throughout the study. For transient transfection, the following genes were cloned into pQCXIH-CMV/TO-DEST (Addgene): FAM86A (UniProt ID: Q96G04) including all FAM86A mutants, FAM86B1 (Uniprot ID: Q8N7N1), FAM86B2 (Uniprot ID: P0C5J1). For bacterial protein expression and purification, FAM86A and its mutants were cloned into pGEX-6P-1 (Addgene). For protein expression and purification from human cells, EEF2 (Uniprot ID: P13639) and its mutants were cloned into pcDNA3.1(+) (Addgene) including the N-terminal FLAG sequence DYKDDDDK.

### Immunoblot analysis

For Western blot analysis, cells were lysed in RIPA buffer with 1 mM PMSF and complete protease inhibitor cocktail. Protein concentration was determined using the Bio-Rad DC Protein Assay. Protein samples were resolved by SDS-PAGE and transferred to a PVDF membrane (0.45 μm). Dot blot analysis was performed by directly loading peptides of the indicated concentrations onto a Nitrocellulose membrane. A volume of 1 μl was loaded for immunoblots and 5 μl was loaded for Ponceau S staining (Sigma). The following antibodies were used at the indicated dilutions: EEF2-K525me3 (1:1000) (Abclonal), EEF2 (1:10,000) (Abcam; ab75748), FAM86A (1:500) (Genemed), tubulin (1:4000) (Millipore; catalog 05-661), FLAG M2 (1:4000) (Sigma-Aldrich; F1804). Mouse and rabbit secondary antibodies from Jackson Immunoresearch were used at 1:10,000 dilution. Protein bands were visualized using Amersham ECL or Amersham ECL Prime Western Blotting Detection Reagent.

### Protein expression and purification

Plasmids encoding FLAG-fusion proteins were transfected into 293T cells selected for CRISPR-mediated FAM86A depletion. Whole cell extracts were prepared in lysis buffer containing 50 mM Tris-HCl (pH 7.5), 250 mM NaCl, 0.5% Nonidet P-40, 10% glycerol, and complete protease inhibitor cocktail 48 h after transfection. Equal amounts of whole cell extracts were incubated with equal volumes of anti-FLAG M2 magnetic beads slurry (Sigma) at 4 °C overnight and either eluted with 0.2 mg/ml 3× FLAG peptide (Sigma) for *in vitro* methylation or resuspended in Laemmli buffer for Western blot analysis.

For crystallography, the DNA encoding full-length human FAM86A was inserted into an in-house bacterial expression vector, in which the FAM86 gene is preceded by an N-terminal hexa-histidine (His6)-MBP tag and a TEV cleavage site. To overcome the challenge of crystallization, three mutations (I256Y, M257Y, and E296Y) were introduced to reduce surface entropy. The expression plasmid was transformed into BL21(DE3) RIL cells. The transformed cells were grown at 37 °C until cell density (*A*_600_) reached 1.0. Protein expression was then induced by addition of 0.13 mM isopropyl β-D-1-thiogalactopyranoside (IPTG), and the cells continued to grow at 16  °C overnight. The cells were harvested and lysed in a buffer containing 50 mM Tris-HCl (pH 8.0), 1 M NaCl, 25 mM Imidazole, 10% glycerol, and 1 mM PMSF. After centrifugation, the fusion protein in the soluble fraction was purified through a nickel column, followed by removal of His6-MBP tag by TEV cleavage, ion-exchange chromatography on a Q HP column (GE Healthcare), and size-exclusion chromatography on a HiLoad 16/600 Superdex 75 pg column (GE Healthcare). The purified protein samples were concentrated in 20 mM Tris-HCl (pH 7.5), 100 mM NaCl, 5% glycerol, and 5 mM DTT and stored at −80 °C.

Plasmids encoding GST-fusion proteins were transformed into BL21 *Escherichia coli* and grown up in LB medium (10 g/L tryptone, 5 g/L yeast extract, and 10 g/L NaCl). Protein expression was induced by 0.1 mM IPTG (isopropyl 1-thio-b-D-galactopyranoside, Sigma) in overnight culture at 18 °C. Proteins were purified using Glutathione Sepharose 4B (GE Healthcare) and eluted in 10 mM reduced glutathione (Sigma). Protein concentrations were measured using Pierce Coomassie Plus Assay, and DTT was added to a concentration of 5 mM.

### Custom antibody generation

The peptide spanning EEF2-Lys525 (VEGL(Kme3)RLAK) was synthesized and purified by high-performance liquid chromatography (>95% purity). Peptides were conjugated to KLH and used as antigen to immunize rabbits. Rabbit protocols, peptide conjugation, immunization, and antiserum production were performed by Abclonal Technology. Antiserum was negatively selected against an identical, unmodified peptide (VEGLKRLAK). Final purification was performed with the immobilized antigenic peptide to select for methyl-specific antibodies. Peptides carrying Kme1 and Kme2 were also synthesized for antibody validation.

The first 126 amino acids of FAM86A were cloned into pGEX-6P-1 and purified as a GST-fusion protein. GST-FAM86A(1–126) protein was then used as an antigen to immunize rabbits for polyclonal antibody production at Genemed Biotechnology, Inc. Antiserum was enriched for specific FAM86A antibodies using NHS-activated High Performance columns (Sigma) following manufacturer’s instructions. Briefly, GST-FAM86A(1–126) was immobilized in coupling buffer containing 200 mM NaHCO3 (pH 8.3) and 500 mM NaCl. Antiserum was diluted 10-fold in dilution buffer containing 10 mM Tris-HCl (pH7.5) and injected over the column. Specific antibodies were collected in elution buffer containing 100 mM Glycine (pH 2.5).

### AlphaFold protein prediction

Individual structures were downloaded either from AlphaFold (https://alphafold.ebi.ac.uk) or AlphaFill (https://alphafill.eu) as indicated. Combinatorial FAM86A–EEF2 structures were determined by querying the FAM86A and EEF2 primary protein sequences (Uniprot) in ColabFold (https://colab.research.google.com) in 1:1 stoichiometry with default parameters. Rank 1 structures were visualized and analyzed with PyMOL after all ranked structures were examined for overall similarity.

### Crystallization and structure determination

For crystallization of the FAM86A–SAH complex, ∼12 mg/ml human FAM86A mixed with 1 mM SAH was incubated with 0.1 M Ammonium citrate tribasic (pH 7.0), 10% w/v Polyethylene glycol 3350, and 5 mM TCEP using the hanging-drop vapor diffusion method at 12 °C. The crystals appeared overnight and continued to grow for 1 week. The crystals were soaked in the crystallization buffer supplemented with 25% (v/v) glycerol before being flash frozen in liquid nitrogen. X-ray diffraction data were collected on the beamline 24-ID-C at Advanced Photo Source, Argonne National Lab. The datasets were processed with the HKL3000 program (36). The structure of the SAH bonded hFAM86A was solved by molecular replacement with the PHASER program (37) using a structural model (ID: AF-Q96G04-F1) predicted by the AlphaFold program (23) as search model. Iterative cycles of model rebuilding and refinement were performed with COOT (38) and PHENIX (39), respectively. The statistics for data processing and structure refinements are summarized in Table S1.

### Protein sequence analysis and alignments

Protein sequences were retrieved under the indicated UniProt IDs and queried using InterPro (https://www.ebi.ac.uk/interpro/) for domain structure analysis or protein BLAST (https://blast.ncbi.nlm.nih.gov/Blast.cgi) for alignments.

### *In vitro* methylation

*In vitro* methylation assays were performed as described (40) by combining up to 3 mg of recombinant proteins in assay buffer containing 50 mM Tris-HCl (pH 8.0), 20 mM KCl, 5 mM MgCl2, and 10% glycerol supplemented with 100 μM *S*-adenosyl-methionine (New England Biolabs) or 2 mCi of tritiated AdoMet (American Radiolabeled Chemicals). The reaction mixtures were incubated overnight at 30 °C. Reactions were resolved by SDS-PAGE and either stained with Coomassie or transferred to a PVDF membrane (0.45 μm) for autoradiography.

### DepMap data

Gene effect data (Project Score, CERES) were downloaded directly (https://depmap.org/portal/download/all/) and visualized using R.

## Data availability

All data supporting the findings of this study are available within the paper and supporting information or otherwise publicly available for download. The crystal structure of FAM86A–SAH has been submitted to the Protein Data Bank (ID: 8FZB). DepMap, AlphaFold, and AlphaFill data are publicly available online for direct download.

## Supporting information

This article contains supporting information.

## Conflict of interest

O. G. is a co-scientific founder, consultant, and stockholder of EpiCypher, Inc, K36 Therapeutics, Inc, and Alternative Bio, Inc.
