# Supplementary figures and images for: The FAM86 domain of FAM86A confers substrate specificity to promote EEF2-Lys525 methylation
Source: J Biol Chem. 2023 May 18;299(7):104842. doi: 10.1016/j.jbc.2023.104842 (PMC10285254; doi:10.1016/j.jbc.2023.104842)

Figure S1.

A

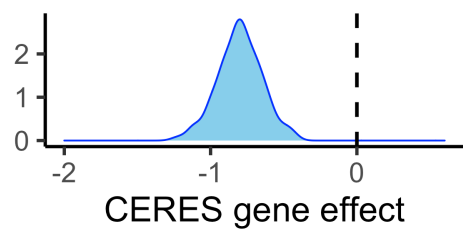

Figure S2.

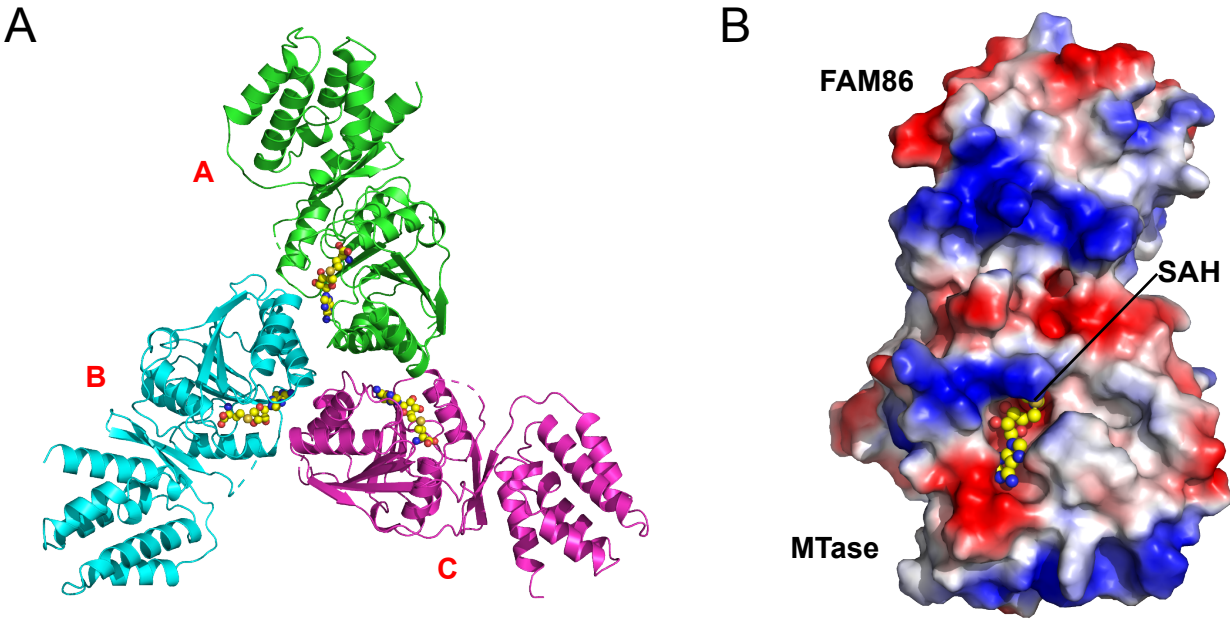

Figure S3.

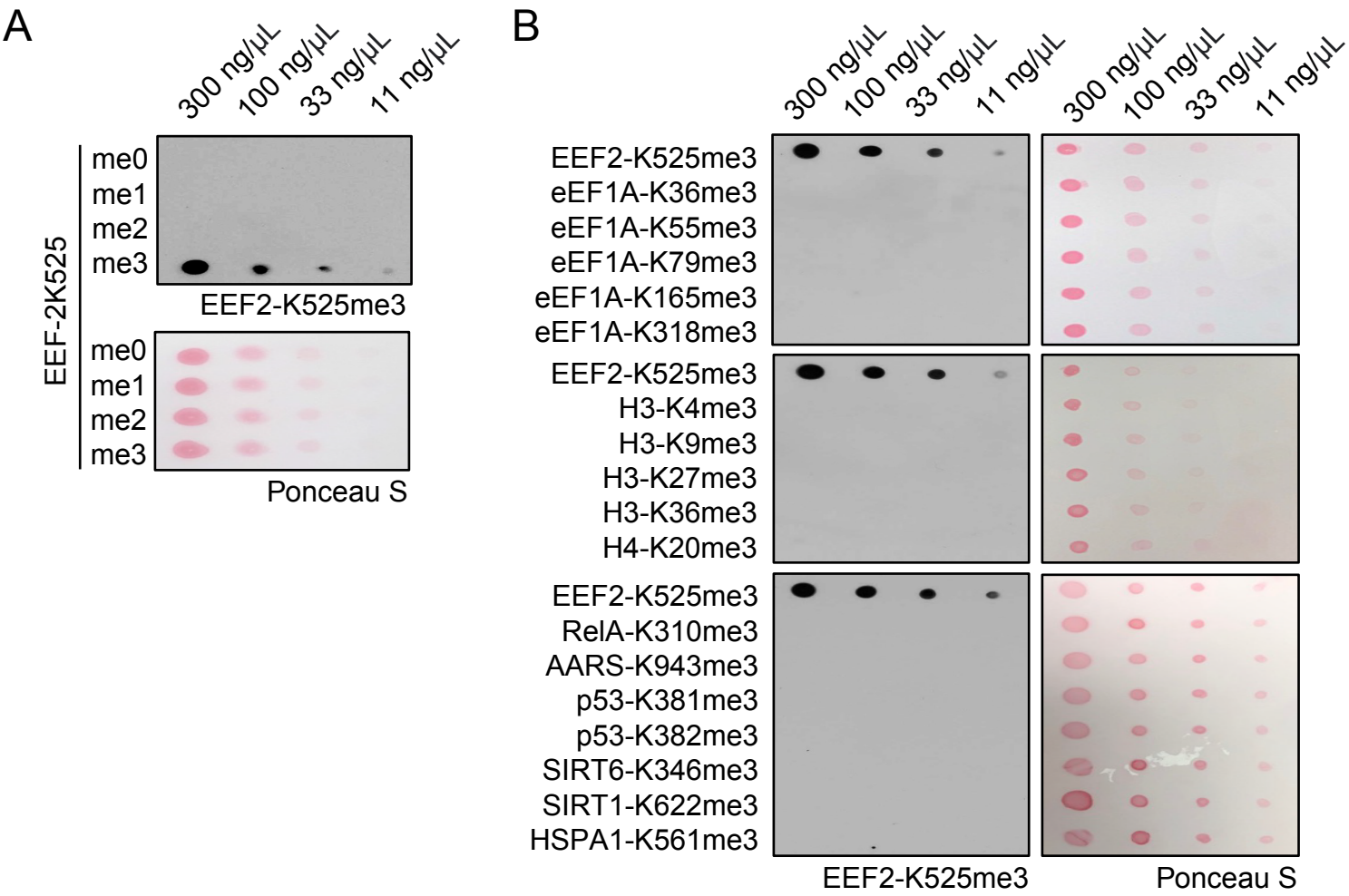

Figure S4.

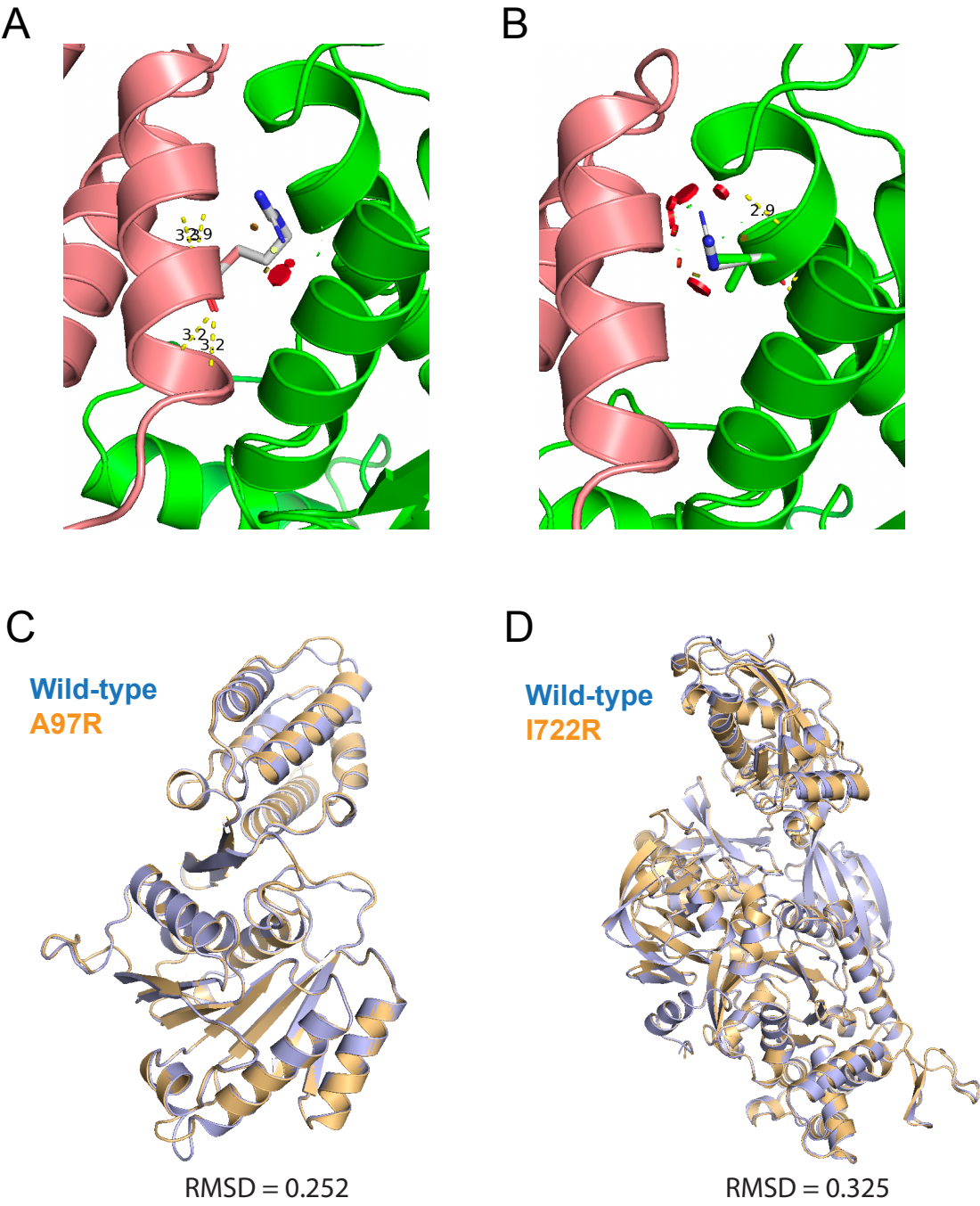

Figure S5.

A

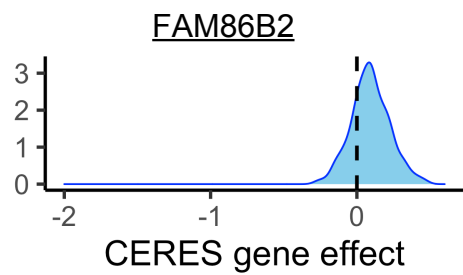

B

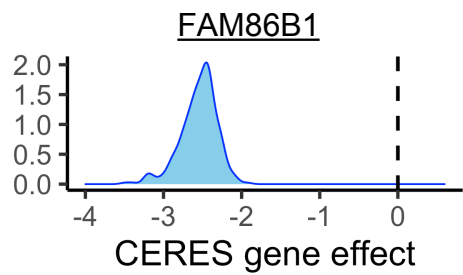

Supplement: Supplemental Figures [file mmc1.pdf]
