# Supplementary material for: The FAM86 domain of FAM86A confers substrate specificity to promote EEF2-Lys525 methylation
Source: J Biol Chem. 2023 May 18;299(7):104842. doi: 10.1016/j.jbc.2023.104842 (PMC10285254; doi:10.1016/j.jbc.2023.104842)
Supplement: Supplementary Figure Legends [file mmc2.docx]

Supplementary Figure Legends

Figure S1. Analysis of CRISPR gene effect scores for FAM86A. (A) Density plot of DepMap gene effect data for FAM86A is shown. Negative CERES scores indicate gene knockout inhibited cell proliferation. FAM86A exhibits an average CERES score near -1, suggesting high dependence of human cancer cell lines on FAM86A expression. n = 317 cell lines

Figure S2. Additional examination of FAM86A crystal structure. (A) Ribbon representation of the three SAH-bound FAM86A molecules in one asymmetric unit, with individual chain IDs labeled using red letters. SAH is shown in sphere representation. Chain A was used for structural analysis.

(B) Electrostatic surface of human FAM86A bound to SAH, with the FAM86 and MTase domains indicated.

Figure S3. Selective and specific recognition of EEF2-K525me3 by custom antibody (A) State-specific dot blot analysis comparing recognition of K525me3 peptides versus K525me0, K525me1, or K525me2.

(B) Protein-specific dot blot analysis comparing recognition of EEF2-K525me3 versus other peptides containing Kme3.

Figure S4. Structural impact of FAM86A(A97R) and EEF2(I722R) mutations. (A) and (B) Simulated effect of FAM86A(A97R) (A) and EEF2(I722R) (B) substitutions on the interface predicted by AlphaFold-Multimer. Close-up view of the FAM86A/EEF2 interface is shown in ribbon representation with mutated residues shown as sticks. FAM86A is colored in pink and EEF2 in green. Mutant arginine is colored in gray. Rotamers with the least steric hinderance are shown.

(C) and (D) Simulated effect of FAM86A(A97R) (C) and EEF2(I722R) (D) substitutions on overall protein folding of FAM86A and EEF2 individually. Wild-type FAM86A and EEF2 AlphaFold models are colored in blue and mutant models shown in orange. RMSD was calculated over 301 aligned Cα atoms for FAM86A and 681 aligned Cα atoms for EEF2.

Figure S5. Analysis of CRISPR gene effect scores for FAM86B2 and FAM86B1 (A) and (B) Density plots of DepMap gene effect data are shown. n = 317 cell lines each.

(A) FAM86B2 exhibits an average CERES score near 0, suggesting low dependence of human cancer cell lines on FAM86B2 expression.

(B) FAM86B1 exhibits an average CERES score less than -2, suggesting high dependence of human cancer cell lines on FAM86B1 expression.
